# Complications associated with the use of temporary pacemaker in patients waiting for definitive device implantation

**DOI:** 10.31744/einstein_journal/2022AO8013

**Published:** 2022-06-21

**Authors:** Silvana Ellen Ribeiro Papp, Aymée Lustosa Nogueira e Torres, Andres Eduardo Larrovere Vasquez, Luciana Gioli-Pereira

**Affiliations:** 1 Hospital Municipal Dr. Moysés Deutsch Hospital Municipal Dr. Moysés Deutsch; Hospital Israelita Albert Einstein, São Paulo, SP, Brazil.; Hospital Israelita Albert Einstein São Paulo SP Brazil

**Keywords:** Pacemaker, artificial, Atrioventricular block, Cross infection, Hospitals, public, Public health, Longht of stay

## Abstract

**Objective:**

To determine the rate of complications associated with the use of temporary pacemakers in patients in the waiting list for the definitive pacemaker implantation in a public hospital located in São Paulo, SP, Brazil.

**Methods:**

Retrospective observational study based on data extracted from medical records of patients admitted to *Hospital Municipal Dr. Moyses Deutsch, Hospital Israelita Albert Einstein* from January 2014 to December 2018. Patients aged 18 years or older, diagnosed with high degree atrioventricular block upon admission and with indications for definitive pacemaker implantation were included. All-cause mortality, clinical and surgical complications and length of hospital stay while waiting for the procedure were defined as primary outcomes.

**Results:**

The sample comprised 66 patient allocated to one of two groups: with and without the need of temporary pacemaker while in hospital (n=45 and n=21, respectively). The rate of complications was higher in patients who used a temporary pacemaker (p<0.001). These included primarily pneumonia (p=0.048) and length of hospital stay (p=0.029).

**Conclusion:**

Patients who required a temporary pacemaker stayed longer in hospital. Longer hospital stay is associated with higher rates of general complications and all-cause mortality.

## INTRODUCTION

Temporary transvenous pacemakers (TTP) have been used since the 1950s and represent an important advancement in emergency services and intensive care therapy.^(1)^ Second and third-degree atrioventricular block is one of the primary indications for pacemaker (PM) insertion. Artificial cardiac stimulation is a relatively simple procedure indicated in irreversible cases. This indication is supported by Brazilian guidelines published in 2007.^(2)^

Although uncommon, complications associated with TTP are potentially severe.^(1)^ The most common complications include electrode displacement and disconnection, bleeding, myocardial perforation, pulmonary embolism, air embolism, arrhythmias, pneumothorax, extracardiac stimulation and infections.^(3,4)^ Complications associated with temporary cardiac stimulation have led many clinicians to elect definitive over temporary PM insertion or to use a permanent transvenous endocardial electrode system.^(5)^ According to data from the 11^th^ world survey of cardiac pacing and implantable cardioverter defibrillators (2009), Brazil is the country with the lowest number of PM insertions per one million inhabitants compared to other Latin American countries.^(6,7)^ In Brazil, a total of 199 mobile cardiac devices were inserted per one million inhabitants, compared to 216 insertions in Chile, 382 in Argentina and 578 in Uruguay.

These data reveal limitations in healthcare access in this patient population, which should be viewed by medical communities and governments as a matter worthy of improvement. The Brazilian 2007 guidelines still emphasize the needed for services that enable the implantation of cardiac devices such as definitive PM.^(2)^ Implantation laboratories can be deployed in surgical or hemodynamic centers equipped with basic monitoring systems, portable or fixed fluoroscopic imaging units and life support equipment for emergencies. In Brazil, most public hospitals are not equipped for PM insertion.

We raised the hypothesis that limited access to definitive PM implantation may lead to longer hospital stay, implying higher costs than the procedure and the device *per se*, and also increase the rate of clinical and surgical complications.

## OBJECTIVE

To investigate complications associated with use of temporary transvenous pacemaker and length of hospital stay prior to definitive pacemaker insertion in a municipal hospital located in the city of São Paulo, SP, Brazil.

## METHODS

### Study design

Observational retrospective study based on the analysis of patients admitted to *Hospital Municipal Dr. Moysés Deutsch, Hospital Israelita Albert Einstein* (HMBM), from January 2014 to December 2018.

### Eligibility criteria

Patients admitted from January 2014 to December 2018 were selected according to the following inclusion criteria: signature of a consent term, 18 years of age or older, diagnosis of high degree atrioventricular block upon admission and referral for definitive PM implantation.

### Ethical aspects

This study was approved by the Ethical and Research Committee of the City Administration of São Paulo (CAAE: 70929317.9.0000.0086, # 2.446.947). Patients included in the study signed an informed consent term, except those who died on the date of data collection.

### Study variables and outcomes

Patient data were extracted from electronic medical records (Medview - AGFA HealthCare). Data included sociodemographic characteristics, medical history, clinical and surgical complications, length of hospital stay until definitive PM insertion and all-cause mortality.

Complications associated with PM insertion procedures or use of a temporary PM and hospital-acquired complications were evaluated. Study outcomes were general complications (device-related or hospital-acquired), use of invasive devices such as probes and catheters, length of hospital stay and all-cause mortality.

### Statistical analysis

Statistical analyses were carried out using SPSS software for Windows, version 22.0 (IBM, Armonk, NY, USA). Continuous variables with normal distribution were expressed as means and standard deviations. Continuous variables with non-normal distribution were expressed as medians and interquartile ranges. Categorical variables were described using absolute numbers and percentages.

Categorical variables were analyzed using the Fisher’s exact test and the χ^2^ test. Continuous variables with normal distribution were analyzed using the unpaired Student’s *t*-test. Continuous variables with non-normal distribution were analyzed using the Mann-Whitney test. A p value of <0.05 was considered significant in comparative analyses.

## RESULTS

Outcomes of 66 patients were analyzed. These patients were allocated to 1 of 2 groups: patients who used a TTP while in hospital (68.2%) and patients who did not (31.8%). The indication of TTP insertion was based on signs or symptoms of low cardiac output in response to bradyarrhythmia. Baseline characteristics did not differ between groups (Table 1).

**Table 1:** Baseline clinical characteristics

Variable	Group		p value
	With TTP n (%)	Without TTP n (%)	
Permanent PM	38 (84.4)	16 (76.2)	0.499^†^
Sex			
Male	21 (46.7)	10 (47.6)	0.942*
Respiratory symptoms	3 (6.7)	1 (4.8)	>0.999^†^
Atrial fibrillation	10 (22.2)	2 (9.5)	0.311^†^
Hypertension	36 (80)	15 (71.4)	0.532^†^
*Diabetes mellitus*	11 (24.4)	4 (19)	0.758^†^
Renal failure	6 (13.3)	5 (23.8)	0.307^†^
Alcoholism	8 (17.8)	2 (9.5)	0.483^†^
Dyslipidemia	10 (22.2)	4 (19)	>0.999^†^
Heart failure	13 (28.9)	4 (19)	0.394*
Chagas disease	12 (26.7)	2 (9.5)	0.195^†^
Hypothyroidism	4 (8.9)	1 (4.8)	>0.999^†^
Smoking	15 (33.3)	6 (28.6)	0.699*

^†^ Fisher exact test; * χ^2^ test.

TTP: temporary transvenous pacemaker; PM: pacemaker.

The rate of general complications (device-related or hospital-acquired) was higher in patients using a TTP (p<0.001; Table 2). Pneumonia (p=0.048), urinary infection (p=0.012) and renal failure (p=0.017) were the most common hospitalization-related complications in these patients (Table 2). Complications attributed to TTP insertion were uncommon.

**Table 2:** Hospital-acquired complications

Variable	Group		
	With TTP n (%)	Without TTP n (%)	p value
Systemic infection	6 (13.3)	0 (0)	0.166^†^
Pneumonia	9 (20)	0 (0)	0.048^†^
Urinary infection	11 (24.4)	0 (0)	0.012^†^
Phlebitis	3 (6.7)	1 (4.8)	>0.999^†^
Arrhythmia	10 (22.2)	1 (4.8)	0.153^†^
Decompensated heart failure	6 (13.3)	0 (0)	0.166^†^
*Delirium*	3 (6.7)	1 (4.8)	>0.999^†^
Endocarditis	2 (4.4)	0 (0)	>0.999^†^
Acute coronary insufficiency	7 (15.6)	2 (9.5)	0.707^†^
Renal failure	20 (44.4)	3 (14.3)	0.017*
Herpes zoster	2 (4.4)	0 (0)	>0.999^†^
Acute gastroenteritis	2 (4.4)	0 (0)	>0.999^†^
Otitis	1 (2.2)	0 (0)	>0.999^†^
Dental abscess	2 (4.4)	0 (0)	>0.999^†^
Pulmonary embolism	3 (6.7)	0 (0)	0.546^†^
Venous thromboembolism	1 (2.2)	0 (0)	>0.999^†^
Death	8 (17.8)	0(0)	0.048^†^
Complications	35 (77.8)	4 (19)	<0.001*
Invasive blood pressure	6 (13.3)	0 (0)	0.166^†^
Central venous catheter	10 (22.2)	0 (0)	0.024^†^
Bladder catheter	15 (33.3)	2 (9.5)	0.039*

^†^ Fisher exact test; * χ^2^ test.

TTP: temporary transvenous pacemaker.

Patients who required a TTP also required invasive devices such as central venous catheter (p=0.024) and bladder catheter (p=0.039) more often (Table 2).

Baseline infectious conditions and renal function parameters (Table 3) did not differ between groups.

**Table 3:** Laboratory parameters upon admission

Variable	Group	Mean	SD	Median	Minimum	Maximum	n	p value
Leukocytes (n/mm^3^)	With TTP	9,851.33	4,034.04	9,240	4,680	26,410	45	0.700*
	Without TTP	9,317.62	3,566.20	8,810	4,470	21,990	21	
CRP (mg/dL)	With TTP	21.85	36.21	11.5	5	226	41	0.211*
	Without TTP	13.57	13.23	6.7	5	42.7	13	
Creatinine (mg/dL)	With TTP	1.42	1.54	1.1	0.4	10.2	45	0.182*
	Without TTP	1.65	3.32	0.9	0.6	16.1	21	

Reference ranges: Leukocytes = 3500 - 11000/mm^3^; CRP = <5mg/dL; Creatinine = <1.2mg/dL.

* Mann-Whitney Test.

TTP: temporary transvenous pacemaker; CPR: C reactive-protein; SD: standard deviation.

All-cause mortality and length of hospital stay were the primary outcomes in this study. Higher death rates (p=0.048) and length of hospital stay (p=0.029) were observed in patients in the TTP group (Tables 2 and 4 respectively).

**Table 4:** Length of hospital stay

Variable	Group	Mean	SD	Median	Minimal	Maximal	n	p value
Age (years)	Temporary PM	70.9	10.9	70	51	96	45	0.786^†^
	Without temporary PM	69.8	16.0	74	41	94	21	
	Total	70.5	12.6	70	41	96	66	
LOS (days)	Temporary PM	27.3	21.6	21	1	98	45	0.029*
	Without temporary PM	16.0	15.3	10	1	65	21	
	Total	23.7	20.4	18.5	1	98	66	

* Mann-Whitney test; ^†^ Student’s *t*-test.

PM: pacemaker; LOS: length of hospital stay; SD: standard deviation.

Of 66 patients in this sample, 12 (18.2%) did not receive a definitive PM due to death or spontaneous reversion of bradyarrhythmia while waiting for the procedure.

## DISCUSSION

In this study, use of a TTP increased the length of hospital stay and all-cause mortality rates. These outcomes were associated with higher rates of general complications, primarily due to longer hospital stay. In a Danish study published in 2012, researchers described a waiting time for PM implantation of up to 4.5 days due capacity constraints. According to authors of that study, ideally waiting time should be less than 24 hours due to potential temporary PM-related complications.^(8)^

Complications observed in this study were more often related to longer hospital stay than to TTP implantation procedure, as shown in table 2. In fact, potentially serious complications, such as bleeding, myocardial perforation, pulmonary embolism, air embolism, arrhythmias, pneumothorax, extracardiac stimulation and infections, were uncommon.^(3,4)^

Mean intensive care unit (ICU) stay of patients using a TTP in this study was 27.3 days. In spite of the scarcity of data, the existing literature is consistent regarding the higher risk of complications in patients using a temporary PM. Therefore, it has been suggested that permanent PM insertion without prior use of a temporary device should be the preferred approach.^(9)^ Unfortunately, in the city of São Paulo (SP) and in other Brazilian regions, this cannot be achieved via the Brazilian Public Health System (SUS - *Sistema Único de Saúde*).

According to national data gathered up to 2014, the number of permanent PM insertions in Brazil is lower than ideal.^(7,10)^ Although up-to-date data are not available for comparison, this study revealed a maximum waiting time for PM insertion of 98 days. This finding is associated with another important factor, namely the cost of longer hospital stay while patients wait for the procedure.

The cost of ICU stay varies according to the level of technology, availability of specialized personnel and appropriate physical environment. For this reason, ICUs are classified as type I, II, and III by the Ministry of Health.^(11)^ The ICU of the public hospital involved in this study is rated level II and has a daily cost of R$ 478,72.^(12)^ Based on the mean ICU stay of patients using a TTP (27.3 days), the estimated cost of treatment would amount to R$ 13.069,00. These findings suggest costs associated with procedure and the permanent device *per se* are lower (and therefore more attractive) than the costs of longer hospital stay.

In fact, according to the administrative rule published by the Ministry of Health, the cost of a PM insertion procedure ranges from R$ 968,77 to R$ 1.730,51 (single- and multi-chamber respectively).^(13)^ The current estimated cost of a single-chamber device is R$ 4.324,34.^(14)^ Therefore, in most cases the cost of ICU stay would exceeded the cost of the permanent implantation procedure, since longer hospital stay is often required. Of note, the cost of emergency services and hospital stay other than ICU stay should also be accounted for.

In summary, this study revealed that the demand for permanent PM insertion in the municipality of São Paulo (SP) is not met by public health services, with significant negative impacts on morbidity and mortality among patients with heart disease and generation of unnecessary costs to the government. This is an important scenario, which must be revisited by health authorities in order to optimize the use of resources and support the provision of integrated health care to the population.

### Study limitations

Lack of a Control Group (without TTP) and need of retrospective consent to participate to comply with ethical and research committee standards adopted in the municipality of São Paulo, SP, Brazil, are major limitations of this study, which translated into a smaller sample size in spite of the large number of patients who met inclusion criteria.

Limitations aside, this study confirmed that the cost of ICU stay while waiting for the procedure is higher than the estimated cost of permanent PM insertion.

## CONCLUSION

Patients who used temporary pacemakers stayed longer in hospital. Longer hospital stay is associated with higher rates of complications and mortality due to a variety of causes. Costs associated with hospital admission and waiting time to permanent pacemaker implantation often exceeds the combined costs of the device and the implantation procedure.
